# Quantifying the Carbon Reduction Potential of Recycling Construction Waste Based on Life Cycle Assessment: A Case of Jiangsu Province

**DOI:** 10.3390/ijerph191912628

**Published:** 2022-10-03

**Authors:** Hongmei Liu, Rong Guo, Junjie Tian, Honghao Sun, Yi Wang, Haiyan Li, Lu Yao

**Affiliations:** 1 School of Transportation and Civil Engineering, Nantong University, Nantong 226019, China; 2 School of Mechanical Engineering, Nantong University, Nantong 226019, China; 3 Engineering Training Center, Nantong University, Nantong 226019, China

**Keywords:** construction waste, recycling, carbon reduction potential, generation, life cycle assessment

## Abstract

The recycling of construction waste is key to reducing waste generation and CO_2_ emissions. This study aimed to develop a quantitative model for analyzing the carbon reduction potential of recycling construction, demolition, and renovation waste (CDRW) in Jiangsu province. The waste generation rate calculation method and nonlinear autoregressive artificial neural network model were used to estimate and predict CDRW generation. The life cycle assessment was performed to calculate the carbon reduction potential of recycling CDRW. In quantifying the carbon reduction potential, not only construction and demolition waste, but also renovation waste was considered for the first time. The results showed that the total carbon reduction potential of recycling CDRW increased from 3.94 Mt CO_2_e in 2000 to 58.65 Mt CO_2_e in 2020. Steel and concrete were the main contributors. By scenario analysis, the carbon reduction potential of fully recycling CDRW in 2020 increased by 37.79 Mt CO_2_e, a growth rate of 64%. The study further predicts future CDRW generation and the corresponding carbon reduction potential. Our conclusions indicate that 245.45 Mt of CDRW will be generated in 2030, and carbon reduction potential may reach 82.36 Mt CO_2_e. These results will help the government manage construction waste better and reach early achievement of the carbon peak target.

## 1. Introduction

In recent years, global warming has become a major environmental challenge to the sustainable development of human society. The high emission of greenhouse gases, made up of mainly carbon dioxide, is an important factor in global warming [1]. According to the data published by the United Nations Environment Program, the construction industry consumes 40% of the energy, emits 38% of the greenhouse gases, and generates 30% of the waste [2]. Therefore, the construction industry is an important source of CO_2_ emissions and solid waste generation [3].

As one of the pillar industries in China, the construction industry plays an important role in promoting economic development. At the same time, it consumes a mass of construction materials leading to huge amounts of CO_2_ emissions [4,5]. It is estimated that China’s construction industry accounts for 25% of total carbon emissions in the country [6]. Reducing carbon emissions from the construction sector could greatly help China achieve its goals of carbon peaking by 2030 and carbon neutrality by 2060, as proposed at the United Nations General Assembly. Jiangsu Province is one of the most advanced provinces and has made a great contribution to China’s construction industry. It ranks first in the country in terms of economic development, number of buildings, and scale of construction [7,8]. Its gross construction product has continued to grow over the years, accounting for 34% of total output in 2020 [9] (http://tj.jiangsu.gov.cn/, accessed on 27 August 2022). The total profits, taxes, and completed areas of construction enterprises are also ranked at the top for the country [10,11]. In addition, construction waste generation in Jiangsu Province has been at the forefront of China [12]. Therefore, it was a practical decision to take Jiangsu province as the research object.

Due to the different levels of development in the construction industry in the provinces of China, studies on carbon emissions at the national level are vulnerable to bias due to ignoring regional variability. Thus, most scholars have used the provincial and municipal levels as targets in their studies and proposed corresponding carbon reduction measures. For example, Padilla-Rivera et al. [13] assessed the carbon emissions of wood-frame residential buildings in Quebec, Canada, and Jafary Nasab et al. [2] studied the carbon footprint of the construction phase of high-rise building construction in Tehran. Many Chinese case studies have investigated provincial and municipal CO_2_ emissions, such as in Wang et al. [14] and Lin et al. [15]. In addition, the provincial level is the unit of decomposition for the national carbon emission targets in China, which is essential for setting and achieving carbon reduction targets [10]. Moreover, statistics in China are based on the collection of provincial data. The data at the provincial level are usually complete and reliable. Therefore, the results of studies conducted at the provincial level are more accurate and more helpful in guiding the reduction of CO_2_ emissions in China as a whole.

Currently, the study of CO_2_ emissions from the construction sector focuses on the analysis of driving factors and measurement of CO_2_ emissions. Regarding CO_2_ emissions, researchers have measured them from buildings, building projects, or the construction industry. Ma et al. [16] estimated carbon emissions from the residential building sector in China, which was expected to reach peak carbon emissions by 2037. In addition, sensitivity analysis concluded that the per capita floor area and energy intensity of municipal residential buildings had the most significant impact on CO_2_ emissions. Hong et al. [17] assessed the carbon emissions of an apartment building project at the construction phase based on three parts: material manufacturing, transportation, and on-site construction. In addition, some researchers further investigated that the emissions of construction materials were related to carbon emissions. Syngros et al. [18] studied the construction materials of Greek houses and estimated the embodied CO_2_ impact on the environment. The results showed that concrete and steel accounted for the largest proportion of CO_2_ emissions. All the above studies were based on the amount of CO_2_ generated by building production activities and did not consider the potential for carbon reduction through the recycling of construction waste.

Construction, demolition, and renovation waste (CDRW), sometimes simply called construction waste, is defined as solid waste generated during the construction, demolition, and renovation of buildings [19,20,21]. Construction and demolition waste have a similar composition to renovation waste in the CDRW, but not in the same proportions [22]. Its sources are wide and complex, mainly including concrete, bricks, asphalt, steel, wood, glass, and ceramics [23]. In recent years, China’s CDRW generation has reached billions of tons, accounting for more than 40% of total municipal waste [24]. With the development of the economy, the CDRW generation continues to grow. Some studies show that most waste disposal methods in developing countries are mainly landfills and dumping, with a very low recycling rate of 8%, whereas the recycling rate in developed countries is as high as 86% [25]. If the waste can be recycled, it will not only save resources, but also effectively reduce CO_2_ emissions. Therefore, estimating the carbon reduction potential of recycling CDRW is crucial for China to achieve its carbon peak target as early as possible.

Life cycle assessment (LCA) is the most common method used to assess the carbon reduction potential of CDRW. For example, Wang et al. [26] used LCA to estimate the carbon emissions of construction waste in Shanghai. Peng et al. [27] used this method to quantify the specific carbon savings potential of recycling construction and demolition waste in the Greater Bay Area of China. This method was used to assess the environmental impact of a product throughout its life cycle [28,29,30]. The LCA has four stages: product, construction process, use, and end-of-life. In addition, an optional stage of reuse, recovery, and recycling of construction materials at the end of life is included [31]. The construction process stage is the entire process of transporting materials to the site and installing them in the building, whereas the use stage is the use, maintenance, repair, renovation, and replacement of the building, as well as operational energy and water use. As recycling CDRW can significantly reduce the extraction of raw materials, there is great potential for carbon reduction in these processes. However, estimating waste generation is an important part of the carbon emissions from CDRW during the LCA. So far, many methods have been developed to estimate CDRW generation: site visits, lifetime analysis, classification system accumulation, variable modeling, and waste generation rate calculation (WGRC) [32,33,34,35]. The first four methods do not apply to this article, due to the lack of statistical data and difficulty in obtaining survey data. In comparison, the WGRC method is widely used to estimate the amount of generated waste and effectiveness of waste management [36]. Cochran et al. [37] used this method to determine the waste generation and composition of residential and non-residential building areas. Domingo et al. [38] also used this method to quantify the amount of waste from 159 detached residential buildings. At the same time, there are many other methods to predict waste generation, which commonly includes multiple linear regression (MLR), grey model (GM), decision tree (DT), nonlinear autoregressive artificial neural network (NARANN), etc. Lu et al. [39] used the four models, MLR, DT, GM, and artificial neural network, to predict construction waste generation in the Greater Bay Area of China, respectively. Sunayana et al. [40] used NARANN to predict the monthly municipal solid waste generation in India. NARANN is an artificial neural network model for predicting one-dimensional time series responses [41]. This model has better predictive performance when the data is limited and nonlinear [40]. In this study, the WGRC method and NARANN model were applied to estimate the CDRW generation in Jiangsu Province.

The purpose of this study was to develop a quantitative model for calculating the specific carbon emission reduction potential of recycling CDRW. It includes three specific aspects: (1) Estimating and forecasting CDRW production; (2) Based on the life cycle perspective, calculating carbon emission reductions from recycling CDRW and analyzing the results; (3) Predicting future carbon emission reduction potential from the recycling of CDRW in Jiangsu Province. The environmental benefits of waste recycling were measured more comprehensively from the perspectives of CO_2_ emissions and CDRW generation. The results of this study will help China and other developing countries to improve waste management strategies and reduce CO_2_ emissions from the waste sector.

## 2. Methodology

**Figure 1:**
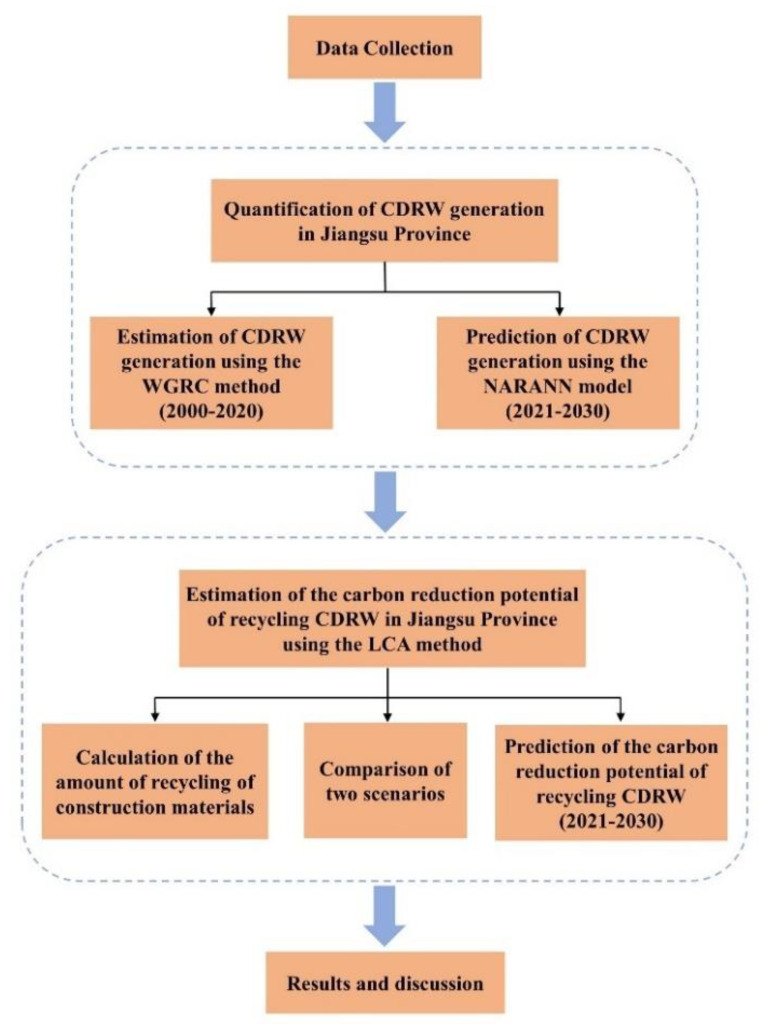
The flow chart of this study (WGRC: waste generation rate calculation; NARANN: nonlinear autoregressive artificial neural network).

This study first estimated CDRW generation in Jiangsu Province, using the WGRC method and NARANN model. Then, we calculated the carbon reduction potential of CDRW recycling in Jiangsu Province from 2000 to 2030, using LCA based on these results. The flow chart of this study is illustrated in Figure 1.

### 2.1. CDRW Generation Estimation

#### 2.1.1. Waste Generation Rate Calculation Method

The waste generation rate calculation (WGRC) method calculates CDRW generation based on the floor area multiplied by the waste generation rate per unit area [39]. In this study, the WGRC method was applied to estimate the CDRW generation in Jiangsu Province in combination with the collected data.

CDRW is composed of construction waste, demolition waste, and renovation waste [19]. The CDRW generation ($W_{total}$) is equal to the sum of these three components, as shown in Equation (1).

(1) $$W_{total}=W_{c}+W_{d}+W_{r}$$

where $W_{c}$, $W_{d}$, and $W_{r}$ are construction waste generation, demolition waste generation, and renovation waste generation, respectively (t). The corresponding calculation equations are listed as the following:

(2) $$W_{c}=A_{c}\cdot P_{c}$$

(3) $$W_{d}=A_{c}\cdot i_{d}\cdot P_{d}$$

(4) $$W_{r}=A_{r}\cdot P_{r}+A_{nr}\cdot P_{nr}$$

where $A_{c}$ is the construction area, (m^2^); $P_{c}$ is the construction waste generation rate per unit area (t/m^2^); $i_{d}$ is the construction demolition area coefficient; $P_{d}$ is the demolition waste generation rate per unit area (t/m^2^); $A_{r}$ and $A_{nr}$ are the completed residential area and completed non-residential area, respectively (m^2^); and $P_{r}$ and $P_{nr}$ are the residential renovation waste generation rate per unit area and non-residential renovation waste generation rate per unit area, respectively (t/m^2^). The CDRW generation rate per unit area and construction demolition area coefficient was obtained by reviewing the literature, as shown in Table 1.

#### 2.1.2. Nonlinear Autoregressive Artificial Neural Network Model

Due to the small amount of data collected in this study, all of them were nonlinear. The nonlinear autoregressive artificial neural network (NARANN) model predicts better under such conditions. Therefore, it was more appropriate to use the NARANN model for prediction. By using MATLAB software, the model was firstly employed to predict the future values of construction, demolition, and renovation waste generated separately. Then, summing up these values gave the future annual generation of CDRW. The mathematical expression of the model is given by Equation (5):

(5) W(t)=f(W(t−1),W(t−2),W(t−3),…,W(t−d))+ε(t)

where W(t) is the data series generated by CDRW varying with time t; f is the activation function used by the artificial neural network model; d is the time delay of the input; and ε(t) is the error approximation of the data series as a function of time.

**Figure 2:**
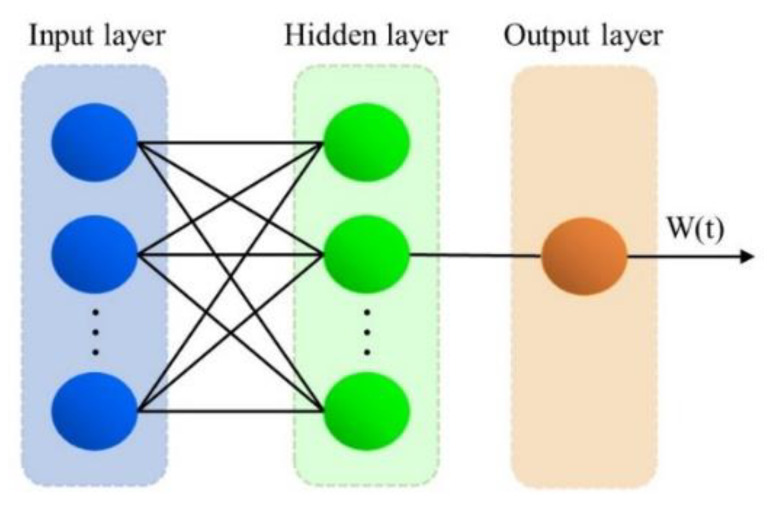
NARANN model structure diagram.

In this study, a NARANN model with four input layers, one output layer, and ten hidden layers was built, and the structure is shown in Figure 2.

To verify the accuracy of the NARANN model, the mean absolute percentage error (MAPE) and the coefficient of determination, $R^{2},$ were used as evaluation criteria [44,45]. The calculation formulas are listed as follows:

(6) $$MAPE=\frac{1}{n}\overset{n}{\underset{i=1}{\sum }}\left|\frac{x\left(t\right)-\hat{x}\left(t\right)}{x\left(t\right)}\right|\times 100\%$$

(7) $$R^{2}=1-\overset{n}{\underset{i=1}{\sum }}\frac{{\left(\hat{x}\left(t\right)-x\left(t\right)\right)}^{2}}{{\left(\bar{x}\left(t\right)-x\left(t\right)\right)}^{2}}$$

where *MAPE* indicates the mean square error between the output and actual value, and the smaller the means the higher the accuracy of the model. $R^{2}$ is a measure of the goodness of fit between the output and actual values. The closer the value is to 1, the better the degree of fit and the more accurate the predicted results.

### 2.2. Assessment of Carbon Reduction Potential from Recycling CDRW

**Figure 3:**
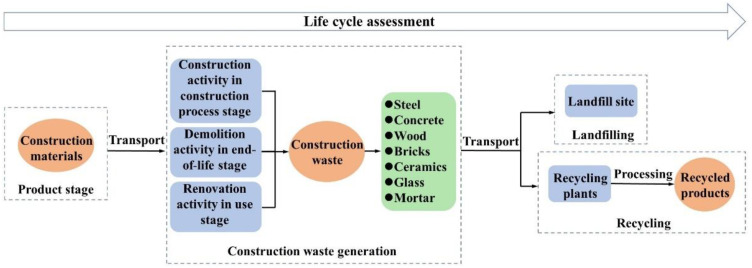
The LCA framework of construction waste.

In the CDRW recycling process, the carbon reduction potential comes from converting the CO_2_ generated in the product and transportation stage into the amount of CO_2_ generated in the recycling process [27]. Several studies have found that concrete, steel, and bricks make up a relatively large proportion of CDRW, and their recycling has high economic value [46]. Therefore, seven major materials in CDRW: steel, concrete, wood, bricks, ceramics, glass, and mortar were selected for the study. The scope of this study included the product, transportation, and recycling stages. The LCA framework of construction waste is shown in Figure 3.

Determining the quantity of material recycled was necessary to calculate the CO_2_ emissions at each stage. The recycling quantity ($Q_{i}$) for each kind of material could be obtained by multiplying three kinds of data: its proportion in construction and demolition waste, its proportion in renovation waste, and its recycling rate, as shown in Equation (8):

(8) $$Q_{i}=\left(W_{c}+W_{d}\right)\cdot CDWR_{i}\cdot RR_{i}+W_{r}\cdot RWR_{i}\cdot RR_{i}$$

where i is one of the seven construction materials; $CDWR_{i}$ is the proportion of material i in construction and demolition waste; $RWR_{i}$ is the proportion of material i in renovation waste; and $RR_{i}$ represents the recycling rate of material i.

The embodied carbon generated by CDRW from cradle-to-site ($E_{cts}$) consists of two parts: the CO_2_ emissions from the product and transportation stage, as shown in Equation (9):

(9) $$E_{cts}=E_{p}+E_{t}$$

where $E_{p}$ represents CO_2_ emissions in the product stage, (kg CO_2_e) and $E_{t}$ indicates CO_2_ emissions in the transportation stage, (kg CO_2_e).

Carbon emissions from the product stage were obtained by multiplying the amount of material recycled by its carbon emission factor. The carbon production in the transportation stage can be calculated by multiplying the values of the quantity of material recycled, average transportation distance, and the carbon emission factor of the transportation tool. The specific formulas are given as follows:

(10) $$E_{p}=\overset{7}{\underset{i=1}{\sum }}Q_{i}\cdot p_{i}$$

(11) $$E_{t}=\overset{7}{\underset{i=1}{\sum }}Q_{i}\cdot D_{i}\cdot t_{i}$$

where i is one of the seven construction materials; $p_{i}$ represents the carbon emission factor of manufacturing material (kg CO_2_e/t); $D_{i}$ represents the transportation distance of material i (km); and $t_{i}$ represents the carbon emission factor of transporting material i (kg CO_2_e/(t·km)).

The carbon emissions from the recycling stage consist of the carbon dioxide emitted by the recycling plants and during transportation to the recycling plants. The calculation formulas are listed as follows:

(12) $$E_{r}=\overset{7}{\underset{i=1}{\sum }}Q_{i}\cdot r_{i}$$

(13) $$r_{i}=2\cdot \bar{D_{r}}\cdot t_{i}+rp_{i}$$

where $E_{r}$ is the carbon dioxide emission in the recycling stage, (kg CO_2_e); $r_{i}$ is the carbon emission factor for recycling material i (kg CO_2_e/t); $\bar{D_{r}}$ is the average distance of material i from transportation to the recycling plant (km); and $rp_{i}$ is the carbon emission factor for processing material i at the recycling plant (kg CO_2_e/t). Based on previous studies, this study assumes that the average transport distance to the recycling plant is 100 km [47].

Finally, the carbon reduction potential of recycling CDRW ($EC_{saved}$) was calculated by Equation (14).

(14) $$EC_{saved}=E_{cts}-E_{r}$$

### 2.3. Data Sources

A large amount of statistical data was collected to estimate the generation of CDRW and the carbon emission saving potential of CDRW recycling. In this study, the data on construction areas, completed residential areas, and completed non-residential areas from 2000–2020 were taken from the Jiangsu Statistical Yearbook published by the Jiangsu Provincial Bureau of Statistics (Table 2) [9] (http://tj.jiangsu.gov.cn/, accessed on 27 August 2022).

Data on the percentage and recycling rate of seven construction materials were obtained based on previous investigations, as shown in Table 3. Material percentage data in construction and demolition waste were supplied by a study on construction and demolition waste generation in China [48]. The data for the percentage of various materials in renovation waste was derived from the amount of commercial housing renovation waste generated in nine cities in the Greater Bay Area of China [49]. Material recycling rate data were sourced from a study by Luo et al. [50]. In addition, it is worth noting that the recycling rate of mortar was set as 8%, which is the average recycling rate of CDRW materials in China [25].

Table 4 shows the parameters of carbon emission reduction potential calculation. The production carbon emission factor, transportation carbon emission factor, and transportation distance of the materials were taken from the standard for calculating carbon emissions from buildings (GB-51366) [51]. Similarly, the missing carbon emission factor of ceramic production in the standard was set at 620 kg CO_2_e/t by Peng et al. [27]. Meanwhile, the materials were mainly transported by medium-duty or heavy-duty diesel trucks. The carbon emission factor for processing materials in recycling plants was also derived from Peng et al. [27].

The recycling rate is an important parameter in assessing the carbon reduction potential of CDRW. To compare the effect of the recycling rate on the reduction of CO_2_ emissions, this study considered two scenarios: the current scenario (where the recycling rate remains constant) and the maximum scenario (where the recycling rate reaches 100%). The carbon reduction potential of recycling CDRW under the two scenarios was calculated and compared. In addition, based on the predicted data of CDRW generation, this study also estimated the carbon reduction potential of CDRW in Jiangsu Province over the next ten years. According to the “Fourteen Five” Circular Economy Development Plan issued by the National Development and Reform Commission in 2021, the comprehensive utilization rate of waste is required to increase from 50% in 2020 to 60% in 2025 [52]. Therefore, the waste recycling rate must increase by 2% per year to improve future carbon reduction potential.

## 3. Results and Discussion

### 3.1. CDRW Generation in Jiangsu Province

**Figure 4:**
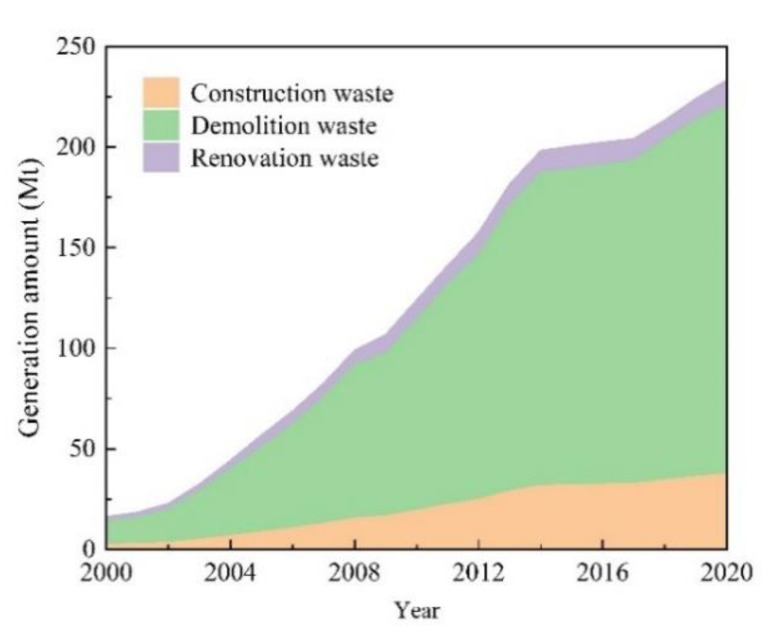
Estimated annual generation of CDRW in Jiangsu Province from 2000 to 2020.

According to the calculation model mentioned above and the collected data, Figure 4 shows the estimated annual generation of CDRW in Jiangsu Province from 2000 to 2020. In terms of the total amount of waste, there is an overall increasing trend. The CDRW generation in Jiangsu province increased from 16.2 Mt in 2000 to 233.23 Mt in 2020, a total increase of 217.03 Mt. The average annual growth rate was 15% in the past 21 years. As the size and number of buildings in Jiangsu Province are growing every year, this leads to the generation of large amounts of CDRW.

In terms of the percentage of CDRW generated, demolition waste generation was the largest, accounting for more than 70% of total waste generation. In contrast, construction and renovation generated waste accounting for 16 and 8% of the total CDRW, respectively. The calculation results show that demolition waste is the primary source of CDRW generation. This can be explained by the fact that the generation rate per unit area of demolition waste is much higher than that of construction and renovation waste. In recent years, Jiangsu Province has been committed to the promotion of old neighborhood renovation projects for better urban development. According to the notice issued by the Housing and Urban-Rural Development Department of Jiangsu Province regarding the renovation of old urban neighborhoods, there are 1405 old neighborhood renovation tasks to be completed in 2022 [53]. This means that CDRW management will continue to face major challenges in the continuous growth of demolition waste.

**Figure 5:**
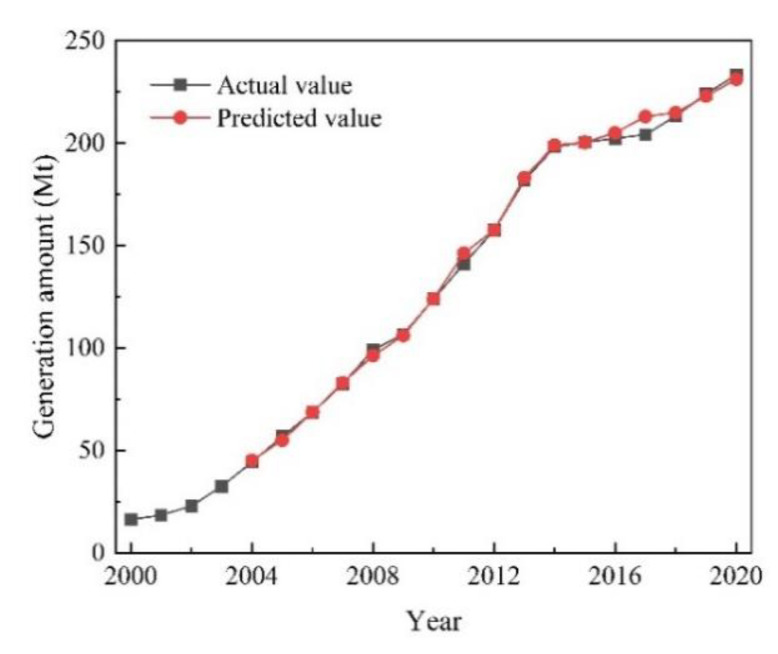
The comparison between the predicted and actual values of CDRW generation.

Figure 5 shows the comparison between the predicted and actual values of CDRW generation. The predicted results had good agreement with the actual values, with $R^{2}$ values over 0.95. Meanwhile, the MAPE values were all less than 3%, which again demonstrates the accuracy of the model.

**Figure 6:**
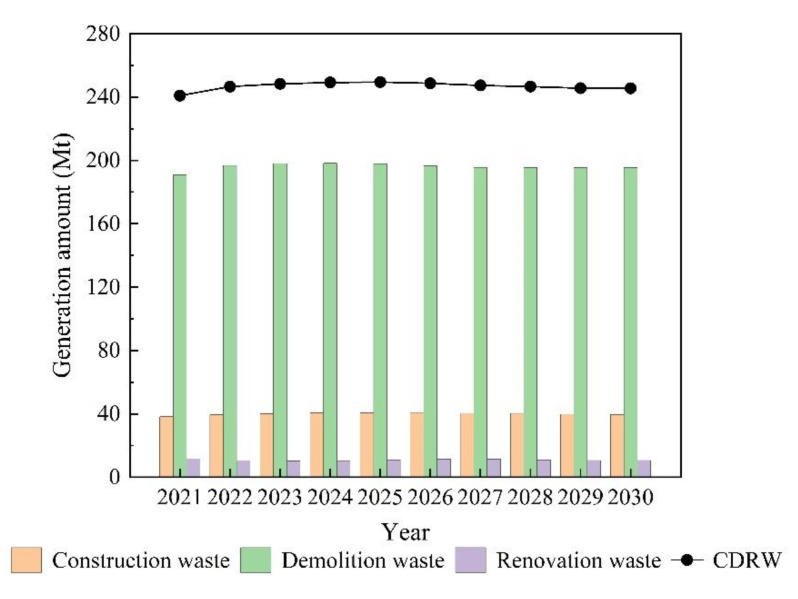
Prediction of annual CDRW generation in Jiangsu Province from 2021 to 2030.

This developed model was used to predict construction waste, demolition waste, and renovation waste generation in Jiangsu Province separately. Figure 6 shows the forecast results of annual CDRW generation in Jiangsu Province from 2021 to 2030. According to the figure, the growth of CDRW generation in Jiangsu Province tends to flatten out in the next decade. By 2030, CDRW generation will reach 245.45 Mt, with an average annual growth rate of 0.21%. However, the overall base is large and still needs reasonable management to reduce waste generation. Improvements in this situation can be achieved in the future by increasing the waste recycling rate. According to the predicted results, there will be a slight decrease in the total amount of CDRW in Jiangsu Province from 2026 to 2030. This can be attributed to the high attention of the government and implementation of a series of waste management measures. Demolition waste still accounts for the largest percentage of the predicted CDRW generation. In the future, CDRW management should focus on reducing the generation of demolition waste.

### 3.2. Carbon Reduction Potential of Recycling CDRW

**Figure 7:**
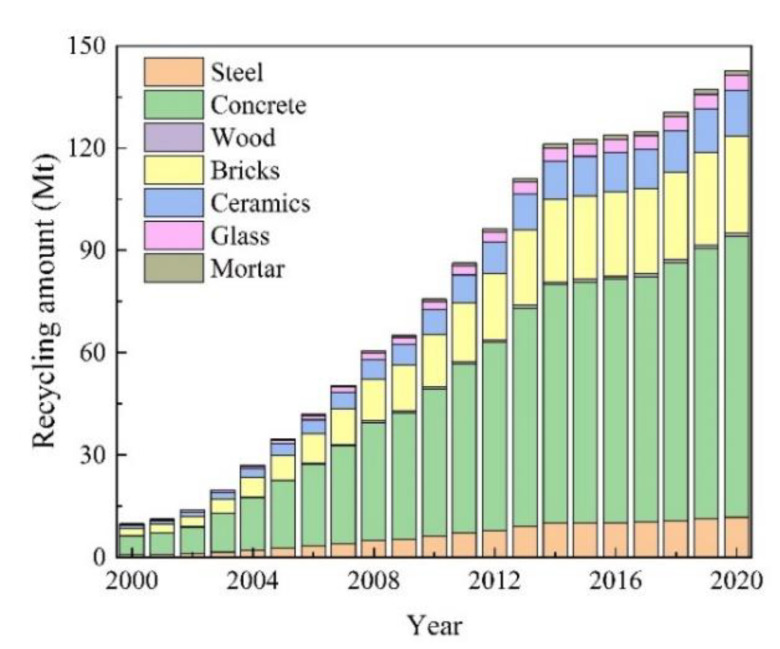
The annual recycling quantity of construction materials in Jiangsu Province from 2000 to 2020.

The calculated annual recycling quantities of construction materials are shown in Figure 7. The results show that the total amount of construction material recycled in Jiangsu province significantly increased from 9.87 Mt in 2000 to 142.72 Mt in 2020, with an average annual growth rate of 14.83%. The growth in the amount of construction material recycled from 2014 to 2017 was slow because the quantity of construction materials recycled is related to the generation of CDRW. With a constant recycling rate, the more CDRW is produced, the more waste recycling occurs. Although the amount of recycling has increased yearly, it is still lower than in other developed countries. According to other studies, 50–80% of all construction waste is reusable or recyclable [54]. Therefore, there is huge potential for CDRW recycling in Jiangsu Province. In terms of the types of materials that were recycled concrete and bricks were the most recycled of all materials. The average annual recycling quantity of the two materials together accounted for 78% of the total construction materials recycled because they could be recycled conveniently and converted into recyclable building materials [55,56]. At the same time, recycling reduces the dissipation of raw materials and benefits the environment.

**Figure 8:**
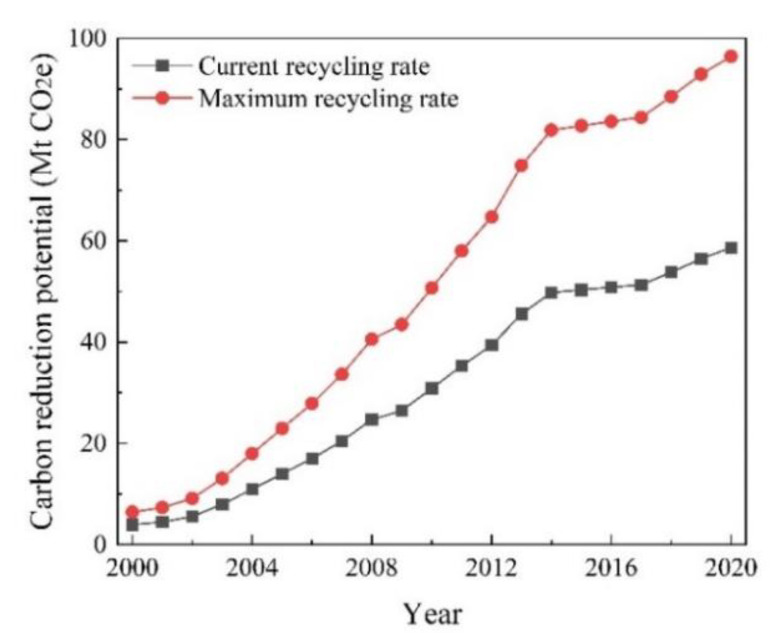
The annual carbon reduction potential of recycling CDRW at different recycling rates in Jiangsu Province from 2000 to 2020.

The annual carbon reduction potential of CDRW recycling in the two scenarios is shown in Figure 8. In the current scenario, where the recycling rate remains constant, the carbon reduction potential increases from 3.94 Mt CO_2_e in 2000 to 58.65 Mt CO_2_e in 2020. In the maximum scenario, where the waste is fully recycled, the carbon reduction potential increases to 96.44 Mt CO_2_e in 2020. Compared with 2020, the carbon reduction potential of fully recycling CDRW increases by 37.79 Mt CO_2_e, with a growth rate of 64%. This is because of the substantial increase in the CDRW recycling rate, which also makes the carbon reduction potential significantly higher.

Table 5 compares the carbon reduction potential of different recycling materials under the two scenarios. In terms of material type, recycling steel and concrete in the current scenario significantly contributes to CO_2_ reduction, accounting for 39.48 and 38.04% of the total carbon reduction potential, respectively. Compared to the current scenario, the contribution of bricks and mortar increases under the recycling rate of 100%, which together account for 27.53% of the total carbon reduction potential. Especially mortar, because it currently has a relatively low recycling rate, the potential for carbon reduction after a large increase is more significant. In addition, steel and concrete continue to be the largest contributors. The study found that most of the carbon emissions from steel come from the product stage. The use of recycled steel emits half the amount of carbon dioxide of virgin steel [57]. Moreover, it has a higher recycling rate and more mature recycling technology [58]. Therefore, the recycling of steel is more prominent in CO_2_ reduction, which is also consistent with the findings of other scholars [26]. Meanwhile, concrete makes up the largest percentage of waste recycling, and after treatment, has huge potential for carbon emission reduction.

An analysis of the total carbon reduction potential of Jiangsu Province shows that an increase in CDRW recycling will bring about considerable environmental benefits. The study shows that the implementation of recycling measures will effectively reduce the carbon dioxide emissions of Jiangsu Province, which has an average annual growth rate of 15%. Moreover, as a large construction province, Jiangsu Province constructs many buildings every year. Taking appropriate recycling measures would have significant environmental benefits, especially in controlling CO_2_ emissions. In addition, using recycled waste in the production process can effectively decrease costs. Recycling enterprises can also supply more jobs to the local area [59]. Therefore, reasonable policies for waste recycling are beneficial to environmental protection and social development.

### 3.3. Future Carbon Reduction Potential Prediction for CDRW Recycling

**Figure 9:**
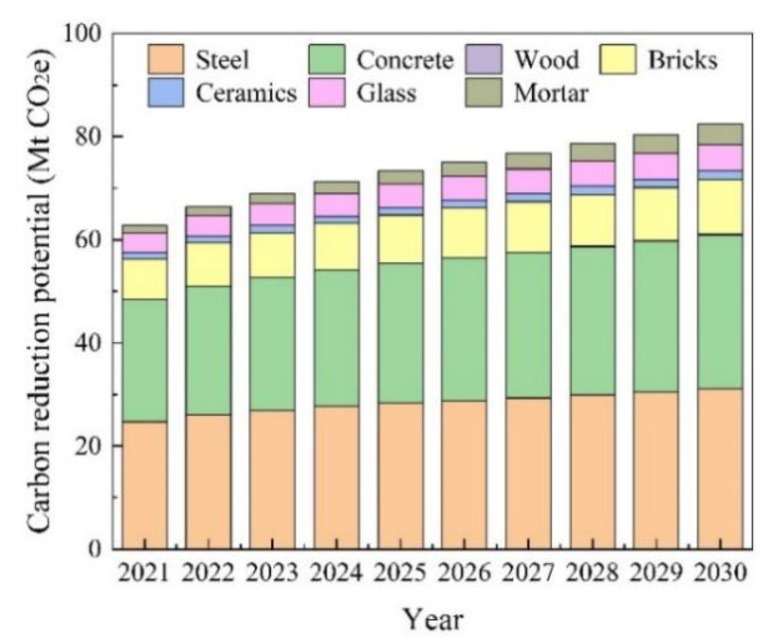
The annual carbon reduction potential of recycling CDRW in Jiangsu Province from 2021 to 2030.

The annual carbon reduction potential of recycling CDRW in Jiangsu Province from 2021 to 2030 is shown in Figure 9. In the context of carbon peaking, the future carbon emission reduction potential was predicted by assuming the recycling rate would increase by 2% per year. The predicted results show that the carbon reduction potential of recycling CDRW is 82.36 Mt CO_2_e by 2030, with an average annual growth rate of 3.08%. From 2021 to 2030, the carbon reduction potential of recycling CDRW in Jiangsu Province shows a slow growth trend in general, which is brought about by the reduction in CDRW generation and an increase in recycling rate. Taking reasonable measures to strengthen the management of CDRW in the future, CO_2_ emissions will continue to decrease.

## 4. Conclusions

As urban development continues and urban construction activities increase, it brings about the generation of large amounts of CDRW. Recycling waste is an effective method for its obvious carbon emission reduction effect. This study quantifies the waste generation and carbon reduction potential from 2000 to 2030 in Jiangsu province. The results show that the total carbon reduction potential increases from 3.94 Mt CO_2_e in 2000 to 58.65 Mt CO_2_e in 2020 with proper waste recycling. Steel and concrete are the main contributors and have the greatest impact on the final carbon reduction potential. This study also conducted different scenario analyses to simulate the carbon reduction potential under two scenarios. Compared with the current scenario, the carbon reduction potential of fully recycling CDRW in 2020 increased by 37.79 Mt CO_2_e, with a growth rate of 64%. It is expected that 245.45 Mt of CDRW will be generated by 2030, with a carbon reduction potential of 82.36 Mt CO_2_e.

According to our study results, it is critical to have effective CDRW management. The government has also recognized the importance of CDRW management, but better policies are needed to deal with the increasing waste. Strengthening technological innovation and research can improve the rate of waste recycling, in particular, of steel and concrete. Promoting the application of recycled products is also an effective measure to reduce waste and increase recycling. The lack of quality standards and the high price of corresponding products have greatly reduced the usage of recycled products. Add to this the fact that the construction industry has only a superficial awareness of the benefits of recycling CDRW. These phenomena require government involvement to improve this situation by strengthening the dissemination of knowledge regarding CDRW recycling and setting mandatory standards. In addition, it is also essential to crack down on illegal practices and strengthen the supervision and inspection of waste management. This could effectively control the generation of waste and its harmful influence on the environment, and thus, achieving sustainable development.

In this study, renovation waste was considered to assess the carbon reduction potential of recycling CDRW, which made our predictions more accurate and had greater reference value. These results could be used to develop more detailed waste management strategies for the government and related departments to achieve the carbon peak target as soon as possible. This quantitative model could also be applied to other countries or regions to estimate carbon reduction potential. However, there were limitations to this study. Due to the unavailability of additional data, only seven major construction materials were studied. Even though there are more types of CDRW, each construction material contributes to CO_2_ emission in some way. In future work, with more data available, the study of more types of construction materials should be added to get a more accurate carbon reduction potential. Moreover, carbon emission reduction potential was also affected by several factors, such as materials and carbon emission factors. Applying advanced modeling methods for analysis could provide an accurate reference for policymakers. Based on the life cycle theory, a comprehensive assessment of the carbon reduction potential of CDRW recycling was beneficial in promoting the low carbon development of the construction industry and achieving energy saving and emission reduction.

## Figures and Tables

**Table 1:** Parameters for construction waste generation estimates.

Parameter	Unit	Value	Reference
Construction waste generation rate per unit area	t/m^2^	0.055	Liu et al. [42]
Demolition waste generation rate per unit area	t/m^2^	1.35	Liang et al. [3]
Residential renovation waste generation rate per unit area	t/m^2^	0.1	Liang et al. [3]
Non-residential renovation waste generation rate per unit area	t/m^2^	0.15	Liang et al. [3]
Construction demolition area coefficient	%	20	Yuan et al. [43]

**Table 2:** Details of the floor area of Jiangsu Province 2000–2020. (unit: million m^2^).

Year	Construction Area	Completed Residential Area	Completed Non-Residential Area
2000	42.68	17.75	3.68
2001	48.58	19.24	4.18
2002	61.16	22.63	4.34
2003	89.25	26.21	5.00
2004	123.16	32.17	6.89
2005	156.19	44.98	10.02
2006	191.08	47.46	11.88
2007	232.22	51.61	11.79
2008	281.88	54.90	12.15
2009	299.54	67.31	17.11
2010	351.07	65.54	21.43
2011	405.00	64.77	19.71
2012	450.98	76.87	21.61
2013	525.74	75.84	21.27
2014	576.38	72.59	23.61
2015	581.18	79.30	23.67
2016	587.62	76.03	24.71
2017	594.64	70.90	24.92
2018	626.73	63.60	21.76
2019	656.87	69.69	24.00
2020	678.89	82.73	28.78

**Table 3:** Percentage and recycling rate of seven construction materials (unit: %).

Material	Construction and Demolition Waste Percentage	Renovation Waste Percentage	CDRW Recycling Rate
Steel	7	2	75
Concrete	48	31	75
Wood	2	3	20
Bricks	21	42	55
Ceramics	10	18	55
Glass	4	0.5	50
Mortar	8	−	8

**Table 4:** Calculation parameters of carbon reduction potential.

Material	Carbon Emission Factor of Product Stage (kg CO_2_e/t)	Distance (km)	Carbon Emission Factor of Transportation Stage (kg CO_2_e/(t·km))	Carbon Emission Factor of Process Stage (kg CO_2_e/t)
Steel	2380	500	0.057	430
Concrete	295	40	0.057	15
Wood	200	500	0.057	190
Bricks	292	40	0.179	1
Ceramics	620	500	0.057	550
Glass	1130	500	0.129	380
Mortar	735	500	0.057	15

**Table 5:** Comparison of the carbon reduction potential of different recycling materials under two scenarios in Jiangsu Province in 2020.

Material	Current Scenario (Mt CO_2_e)	Percentage (%)	Maximum Scenario (Mt CO_2_e)	Percentage (%)
Steel	23.16	39.48	30.88	32.02
Concrete	22.31	38.04	29.75	30.84
Wood	0.03	0.04	0.13	0.13
Bricks	7.45	12.70	13.54	14.04
Ceramics	1.17	1.99	2.12	2.20
Glass	3.51	5.98	7.01	7.27
Mortar	1.04	1.77	13.01	13.49
Total	58.65	100	96.44	100

## Data Availability

Data will be made available from the corresponding authors on reasonable request.
